# P-251. Assessing the Persistent Effects of an Extensive Hospital Cleaning Initiative on Healthcare-Associated Infections and Multidrug-Resistant Microorganism Colonization in Patients: A Longitudinal Study

**DOI:** 10.1093/ofid/ofae631.455

**Published:** 2025-01-29

**Authors:** Edna M M Leite, Bráulio R G M Couto, Karla Neiva, Eliane Costa, Wagner Luiz De Oliveira, Barbara Luisa Sales, Gleiciane Marcelina Teixeira, Lívia Miranda

**Affiliations:** Hospital Risoleta Tolentino Neves, Belo Horizonte, Minas Gerais, Brazil; AMECI – Associação Mineira de Epidemiologia e Controle de Infecções, Belo Horizonte, Minas Gerais, Brazil; Hospital Risoleta Tolentino Neves - HRTN, Belo Horizonte, Minas Gerais, Brazil; Hospital Risoleta Tolentino Neves - HRTN, Belo Horizonte, Minas Gerais, Brazil; Hospital Risoleta Tolentino Neves - HRTN, Belo Horizonte, Minas Gerais, Brazil; Hospital Risoleta Tolentino Neves - HRTN, Belo Horizonte, Minas Gerais, Brazil; Hospital Risoleta Tolentino Neves - HRTN, Belo Horizonte, Minas Gerais, Brazil; Faculdade Dinâmica Vale do Piranga - FADIP, Viçosa, Minas Gerais, Brazil

## Abstract

**Background:**

Healthcare-associated infections (HAI) pose a significant global threat to patient safety. The hospital environment's role in infection prevention and control has gained renewed importance.

**Objective:** to assess the impact of hospital environmental interventions on improving healthcare environmental hygiene and ultimately reducing patient colonization with multidrug-resistant microorganisms (MDROs) and other epidemiologically significant pathogens.

Secular time trend of monthly incidence density per 1,000 patient-days for nine MDROs, 2021-2022

**Figure:**
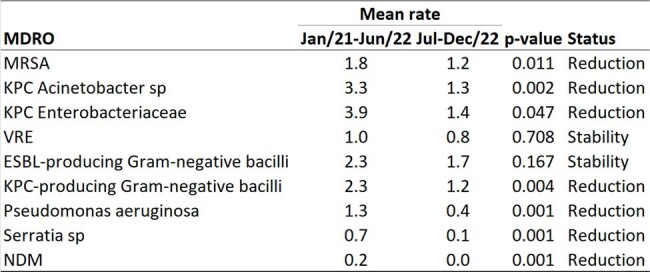

Secular time trend of monthly incidence density per 1,000 patient-days for nine MDROs, 2021-2022

**Methods:**

In 2019, a multidisciplinary environmental organization committee was formed, including representatives from the hospital board, hospitality, occupational health and safety, and pharmacy. This committee launched a dedicated weekly task force to focus on cleaning and integrated surface disinfection throughout the hospital, encompassing all units, including the intensive care units. Regular rounds were conducted by the task force to ensure comprehensive environmental hygiene. The study was conducted at a public institution in Belo Horizonte, Brazil, which has a capacity of 420 beds. The incidence density per 1,000 patient-days was calculated to assess the time trend of nine MDROs across the entire hospital. The MDROs included in the analysis were MRSA, carbapenem-resistant Acinetobacter sp, carbapenem-resistant Enterobacteriaceae, VRE, ESBL-producing Gram-negative bacilli, KPC-producing Gram-negative bacilli, Pseudomonas aeruginosa, Serratia sp, and NDM-producing Gram-negative bacilli.

Monthly incidence density per 1,000 patient-days, and six-month moving average incidence for MRSA, 2021-2022

**Figure:**
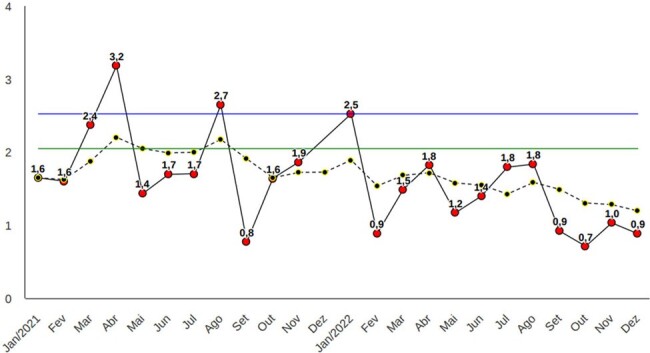

Monthly incidence density per 1,000 patient-days, and six-month moving average incidence for MRSA, 2021-2022

**Results:**

Over the course of four years (2019-2022) of implementing the weekly cleaning task force, we observed a sustained downward trend in the monthly and six-month moving average incidence rates of seven out of the nine MDROs assessed.

Monthly incidence density per 1,000 patient-days, and six-month moving average incidence for carbapenem-resistant Acinetobacter sp, 2021-2022

**Figure:**
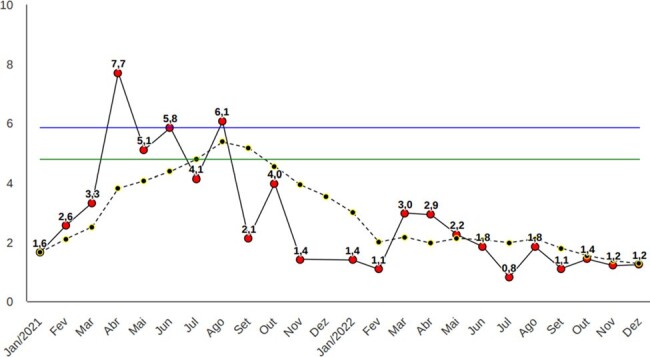

Monthly incidence density per 1,000 patient-days, and six-month moving average incidence for carbapenem-resistant Acinetobacter sp, 2021-2022

**Conclusion:**

Long-term implementation of a dedicated and extensive hospital cleaning task force has resulted in a consistent reduction in MDRO occurrences, demonstrating the effectiveness of this approach.

Monthly incidence density per 1,000 patient-days, and six-month moving average incidence for carbapenem-resistant Acinetobacter sp, 2021-2022

**Figure:**
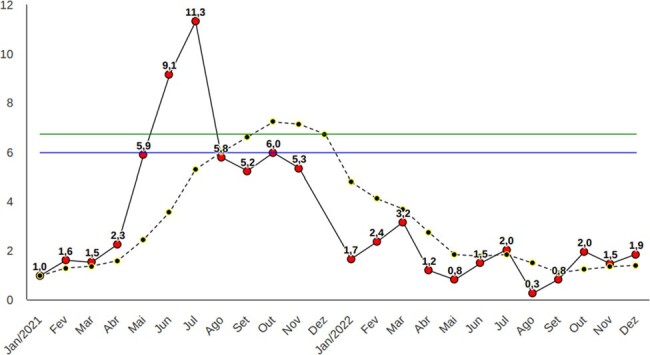

Monthly incidence density per 1,000 patient-days, and six-month moving average incidence for carbapenem-resistant Acinetobacter sp, 2021-2022

**Disclosures:**

**All Authors**: No reported disclosures

